# A multi-country survey on the impact of COVID-19 on dental practice and dentists’ feelings in Latin America

**DOI:** 10.1186/s12913-022-07792-y

**Published:** 2022-03-25

**Authors:** Rafael R. Moraes, Carlos E. Cuevas-Suárez, Wilfredo G. Escalante-Otárola, María R. Fernández, Andrés Dávila-Sánchez, Patricia Grau-Grullon, Eduardo Fernández, Tania M. López, Guillermo Grazioli, Luis A. Arana, Luis F. Rondón, Willy B. Torrez, Giana S. Lima, Helena S. Schuch, Marcos B. Correa, Flavio F. Demarco

**Affiliations:** 1 Universidade Federal de Pelotas, Rua Gonçalves Chaves 457, Pelotas, RS 96015-560 Brazil; 2 Universidad Autónoma del Estado de Hidalgo, San Agustin Tlaxiaca, Mexico; 3 Universidad Catolica de Santa Maria, Arequipa, Peru; 4 Universidad Autónoma de Asunción, Asunción, Paraguay; 5 Universidad San Francisco de Quito USFQ, Quito, Ecuador; 6 Universidad Iberoamericana, Santo Domingo, Dominican Republic; 7 Universidad de Chile, Santiago, Chile; 8 Universidad Autónoma de Chile, Santiago, Chile; 9 Universidad Nacional Autónoma de Nicaragua, Managua, Nicaragua; 10 Universidad de la República, Montevideo, Uruguay; 11 Universidad Santiago de Cali, Cali, Colombia; 12 Universidad de Los Andes, Mérida, Venezuela; 13 Universidad Privada Franz Tamayo, La Paz, Bolivia

**Keywords:** Dental care, Personal protective equipment, COVID-19 testing, Fear, Professional practice

## Abstract

**Background:**

The COVID-19 pandemic has significantly influenced the routine of healthcare workers. This study investigated the impact of the pandemic on dental practice and dentists’ feelings in Latin America.

Methods: A survey was conducted with dentists from 11 Spanish-speaking Latin American countries in September–December 2020. Professionals were invited by email and via an open campaign promoted on social media. The questions investigated dental care routines, practice changes, and feelings about the pandemic. Descriptive statistics were used to identify frequencies and distributions of variables. Proportions were compared using chi-square tests.

**Results:**

A total of 2127 responses were collected from a sample with diverse demographic, sex, work, and education characteristics. The impact of COVID-19 was considered high/very high by 60% of respondents. The volume of patients assisted weekly was lower compared with the pre-pandemic period (mean reduction = 14 ± 15 patients). A high rate of fear to contracting the COVID-19 at work was observed (85%); 4.9% of participants had a positive COVID-19 test. The main professional challenges faced by respondents were reduction in the number of patients or financial gain (35%), fear of contracting COVID-19 (34%), and burden with or difficulty in purchasing new personal protective equipment (22%). The fear to contracting COVID-19 was influenced by the number of weekly appointments. A positive test by the dentists was associated with their reports of having assisted COVID-19 patients. The most cited feelings about the pandemic were uncertainty, fear, worry, anxiety, and stress. Negative feelings were more prevalent for professionals who did not receive training for COVID-19 preventive measures and those reporting higher levels of fear to contract the disease.

**Conclusion:**

This multi-country survey indicated a high impact of the pandemic on dental care routines in Latin America. A massive prevalence of bad feelings was associated with the pandemic.

**Supplementary Information:**

The online version contains supplementary material available at 10.1186/s12913-022-07792-y.

## Background

The coronavirus disease (COVID-19) pandemic has significantly impacted the dental sector worldwide. Studies conducted in several countries have shown a wide range of repercussions in dentistry that included lockdowns limiting access of patients to services, drops in income of dentists, changes in use, cost, and availability of personal protective equipment (PPE), increased patient anxiety levels, changes in the use of emergency services, and dental personnel fear of contracting the disease at work [1–9]. Dentistry is a high-risk profession for COVID-19 as the oral cavity is an important site for severe acute respiratory syndrome coronavirus 2 (SARS-CoV-2) infection and saliva may be a source of contamination [10]. The inapplicability of social distancing and masks for patients is aggravated by the aerosol that can be generated in intraoral procedures. This high-risk scenario may pose psychological challenges to dental personnel [11]. Dentists have been reported to have high rates of fear of contracting COVID-19 at work and elevated levels of psychological distress [2, 7, 12].

In 2020 the COVID-19 outbreak was declared a pandemic, and dental practices in several countries were closed except for emergencies [1, 2, 7]. Since dental care is an essential service, offices were later reopened and have been able to remain working. Latin America has been of the most significant pandemic epicenters, daily accounting for thousands of new cases and deaths [13]. Although the region is known for its large inequalities, it has not usually been the focus of studies on the pandemic impact on dentistry. A study with over 3000 dentists, during the first wave in Brazil, the largest country in the region, reported differences in coverage between public and private dental care networks and new clinical routines for dentists associated with an economic burden on dental practices [7]. Another study in Brazil revealed that although the evolution of COVID-19 among oral health professionals was similar to that of the general population, the cumulative incidence was 5% higher among dental personnel [14]. The scenario in other Latin American countries has not received much attention, and it would help to understand the scale and nature of the impacts on dentistry in the region. A survey with dentists and dental students from Latin American and Caribbean reported influence of the pandemic on social, mental health, and labor aspects of dental staff [15]. In another study, dentists from Latin America and the Caribbean reported that they had assisted patients with COVID-19 more often than the rates of frequency reported for this purpose by their counterparts in Europe [16].

A significant challenge in the ongoing pandemic has been to reach participants willing to engage in clinical or epidemiological research as social distancing is still an effective measure for reducing the chances of contracting COVID-19 [17]. As a result, a pandemic of online research has been described, with methodological issues that have sometimes been overlooked by researchers [18]. The overload of online questionnaires may reduce the interest of potential participants and instigate further challenges to online research. In the present study, the impact of COVID-19 pandemic on dentists in Latin America was investigated by means of an online survey with professionals working in 11 Spanish-speaking countries in the region. The specific objectives of this study were to investigate i) professional practices and dental office routines during the pandemic; ii) COVID-19 contraction rates among dentists and their main professional challenges; and iii) dentists’ feelings about the pandemic.

## Methods

### Study design and ethical aspects

This multi-country cross-sectional survey was implemented in 11 Spanish-speaking Latin American countries between September and December 2020. The participating countries were Bolivia, Chile, Colombia, Dominican Republic, Ecuador, Mexico, Nicaragua, Paraguay, Peru, Uruguay, and Venezuela. The study protocol was approved by an institutional research board from each participating country. All research methods were performed in accordance with the Declaration of Helsinki [19]. The primary objective of the survey was to assess the impact of COVID-19 pandemic on the dental care routines and associated aspects. A questionnaire that had been developed and pre-tested in a previous study [7] was translated to Spanish, adapted to, and pre-tested again for the present investigation. All participants provided informed consent to participate in the study: the respondents had to agree with their participation to access the questionnaire and were directed to print or save the first page of the questionnaire to retain a copy of the consent form. In accordance with open science practices, the research project, the questionnaire in its original language, and the databank of responses have been published in an open platform (doi:10.17605/OSF.IO/DNBGS). An English translation of the questionnaire is presented in the Additional file 1. The Consensus-Based Checklist for Reporting of Survey Studies (CROSS) [20] was consulted for this report.

### Questionnaire development and pre-testing

A self-administered electronic questionnaire was designed to provide data on possible changes in clinical routines and the behavior of dentists during the pandemic. Detailed description of the original questionnaire development can be found elsewhere [7, 21]. For the present study, the questionnaire was translated into and adapted based on the inputs of at least four researchers in three rounds of discrete reviews. The questionnaire was hosted on Google Forms (Google Inc., Mountain View, CA, USA) and pre-tested using a sample of 35 dentists from all the 11 participating countries. There were differences in sex, age, working sector, experience, and education levels across the pre-testers, who evaluated reliability and face validity, writing, and internal consistency. The pre-testers scored the clarity of each question between 1 (not clear) and 5 (very clear) on a Likert scale and were able to explain their scores. No questions were rated with scores 1 or 2; questions rated with at least one score 3 (*n* = 9) were discussed by three or more researchers and edited based on the pre-tester comments. The average ± standard deviation (SD) scores were 4.77 ± 0.09 for the 9 questions requiring revision and 4.86 ± 0.09 for all 32 questions. The pretest was important for the purpose of including other response options, which helped with reducing response bias. A final revision of the questionnaire was carried out iteratively. Pre-testers were excluded from the definitive study.

### Questionnaire content

The initial section of the questionnaire contained the title and main objective of the study, an invitation was extended only to dentists to participate and to complete the questionnaire only once. Informed consent was provided in the second section, in which the participants were notified that their participation was voluntary and not paid, potential risks and benefits were informed, and they were assured that all responses would be treated confidentially and anonymously. The structured questionnaire contained 32 mandatory items (1 open, 31 close-ended questions) divided into four sections (screens): demographic and professional profile (*n* = 11); professional practices during the pandemic (*n* = 10); structure and routine of the respondent’s main workplace (*n* = 7); and a final section (*n* = 4) addressing the respondent’s feeling about the pandemic. No randomization of items or adaptive questioning methods were used. The responses could be reviewed by using back buttons before submitting the form. The main outcomes were related to the professionals’ behavior regarding their clinical routines. The options ‘I’d rather not say’, ‘I don’t know how to answer’, and ‘Does not apply’ were available to avoid response errors. In an open question, the respondents were asked to use a single word to describe a feeling that they would associate with how they felt during the COVID-19 pandemic.

### Sample selection, sample size estimation, and collection of responses

All dentists practicing in the 11 Latin American countries participating in this study were eligible. Researchers from each country contacted local national dental councils to enable the number of registered practicing dentists to be estimated. Given a rough estimation of a target population of approximately 300,000 professionals, we estimated that ~ 1529 responses would be necessary to ensure a 95% confidence interval and 2.5% margin of error. The sample was, however, not expected to be representative of each country but rather to provide a general overview of the impact on dentistry in the region. Responses were collected between September 22 and December 13, 2020.

### Participant recruitment and survey administration

The strategy for recruiting participants began with emails or WhatsApp messages being sent to registered dentists (convenience sampling) and, later, included an open social media campaign targeting dentists in the participating countries. Survey administration was the same irrespective of the mode of invitation since all led the respondent to a unique website link. We tested whether the questionnaire could be read well on different computers, tablets, and cell phones. Approximately 24,500 e-mail invitations and WhatsApp messages were sent via professional dental/health entities or researchers based in each country, and contained a brief statement that included the study objective, the average response time (7 min), and the website link to the questionnaire. Reminder emails and messages were sent after at least 2 weeks. The open social media campaign followed a similar approach to that previously reported [7, 21] and included invitations posted by the research team on Facebook, Instagram, and/or Twitter social networking services. Dentists with professional social media profiles from the 11 participating countries were asked to extend the promotion and contribute to divulging the invitation in their own social media profiles.

### Data analysis

Partial questionnaire completion was not possible. In some questions, the responses were restricted to a specific population, e.g., only dentists assisting patients at the time the survey was responded. Weighting of items or propensity scores were not used. The responses ‘I’d rather not say’, ‘I don’t know how to answer’, and ‘Does not apply’ were considered missing data, resulting in variable numbers of respondents for different questions. Words used in the open question about the feeling associated with the pandemic were translated to English. Singular and plural words, lowercase and capital letters were grouped together. Nouns and adjectives also were grouped for consistency, e.g., anxious/anxiety. The decision was to keep either the noun or adjective considering the most frequent form used. The words were categorized as negative, neutral, or positive feelings. Descriptive statistics were used to identify frequencies and distributions of variables. Proportions were compared using chi-square tests. The numbers of weekly patients assisted before and during the pandemic were analyzed using a paired t-test with unequal variances (α = 0.05). All analyses were performed in Stata 14.2 (StataCorp, College Station, TX, USA).

## Results

A total of 2217 valid responses were received with no atypical timestamp observed. The response rate was ~ 3.2% considering email invitations and WhatsApp messages only, but the numbers of dentists reached, rejections, and losses could not be calculated precisely because different means were used to recruit participants, including a social media campaign. Table 1 presents the demographic and work practice characteristics of the respondents. The study recruited participants from all of the 11 countries, the respondents were of a mean age of 38 ± 11 years, the majority were female (76%), and had been in dental practice for up to 19 years (74%). The majority of respondents reported that they received WhatsApp messages or email invitations to contribute to the survey (81%). Facebook and Instagram social media services accounted for 18% of the invitations received by respondents, whereas Twitter was hardly reported as a means of invitation (< 1%). Most respondents claimed that they worked in the private sector (81%), while the most common postgraduate education they had completed was residency or advanced special training (39%).

**Table 1:** Demographic characteristics of the respondents, Latin America, 2020 (*N* = 2127)

Variable/category	n^a^	%	95% CI
**Sex**	**2125**		
Female	1618	76.1	74.3–77.9
Male	507	23.9	22.1–25.7
**Country**	**2127**		
Bolivia	54	2.5	1.9–3.3
Chile	54	2.5	1.9–3.3
Colombia	73	3.4	2.7–4.3
Dominican Republic	234	11.0	9.7–12.4
Ecuador	87	4.1	3.3–5.0
Mexico	432	20.3	18.7–22.1
Nicaragua	85	4.0	3.2–4.9
Paraguay	183	8.6	7.5–9.9
Peru	157	7.4	6.3–8.6
Uruguay	376	17.7	16.1–19.4
Venezuela	392	18.4	16.8–20.1
**By what means did you receive the invitation to participate in the survey?**	**2127**		
WhatsApp	877	41.2	39.2–43.3
Email	834	39.2	37.2–41.3
Facebook	228	10.7	9.5–12.1
Instagram	154	7.2	6.2–8.4
Twitter	19	0.9	0.1–1.4
Other/I don’t know	15	0.7	0.1–1.2
**Years in practice**	**2109**		
< 10	874	41.4	39.3–43.6
10 to 19	682	32.3	30.4–34.4
20 to 29	344	16.3	14.8–18.0
30 or more	209	9.9	8.7–11.3
**Postgraduate education (completed)**	**2100**		
None	499	23.8	22.0–25.6
Short-term courses	506	24.1	22.3–26.0
Residency or advanced special training	817	38.9	36.8–41.0
MSc or PhD	278	13.2	11.9–14.8
**Main work sector**	**2090**		
Public	241	11.5	10.2–13.0
Private	1687	80.7	79.0–82.4
Other	162	7.8	6.7–9.0

^a^Varies from total N because of missing data for different questions

*CI* Confidence interval

### Professional practices and dental office routines during the pandemic

As shown in Table 2, the volume of patients assisted weekly compared with the pre-pandemic period was reported to be reduced by 88.4% of the respondents, including 5.6% that were not seeing a single patient in the period. Interestingly, 6.1% of the sample reported increased volume of patients during the pandemic. Before the pandemic, the mean ± SD of weekly appointments reported was 25 ± 22 (median = 20), whereas during the pandemic the mean was 14 ± 15 (median = 9), with a difference between the two periods (*P* < 0.001). The impact of the pandemic on work routines was considered high or very high by 60.4% of respondents. In total, 46.1% of the participants reported having had online patient appointments during the pandemic, whereas 39.2% were not willing to conduct online appointments, and 6.9% reported an overall negative experience this format. The masks most frequently worn by respondents when assisting patients were PFF2/N95 (41.6%), disposable surgical mask (27.2%), and surgical mask on top of PFF2/N95 (20.6%).

**Table 2:** Work practice characteristics of the respondents, Latin America, 2020 (*N* = 2127)

Variable/category	n^a^	%	95% CI
**Volume of weekly patients before the pandemic**	**1996**		
0	1	0.05	0.01–0.3
1 to 10	563	28.2	26.3–30.2
11 to 40	1109	55.6	53.4–57.7
41 or more	323	16.2	14.6–17.9
**Volume of weekly patients during the pandemic**	**1996**		
0	112	5.6	4.7–6.7
1 to 10	1095	54.9	52.7–57.0
11 to 40	704	35.3	33.2–37.4
41 or more	85	4.3	3.4–5.2
**Volume of weekly patients compared with pre-pandemic period**	**1940**		
Same	107	5.5	4.6–6.6
Increased	118	6.1	5.1–7.2
Reduced	1715	88.4	86.9–89.7
**Impact of pandemic in work routine**	**2111**		
No impact	40	1.9	1.4–2.6
Low	220	10.4	9.2–11.8
Intermediate	578	27.4	25.5–29.3
High	797	37.8	35.7–39.8
Very high	476	22.6	20.8–24.4
**Have you had online patient appointments during the pandemic?**	**2025**		
No but I am willing to make them	297	14.7	13.2–16.3
No and I am not willing to make them	794	39.2	37.1–41.4
Yes, the overall experience was positive	794	39.2	37.1–41.4
Yes, the overall experience was negative	140	6.9	5.9–8.1
**Do you fear to contract COVID-19 at work?**	**2101**		
No	324	15.4	13.9–17.0
Yes, a little	517	24.6	22.8–26.5
Yes, moderately	953	45.4	43.2–47.5
Yes, a lot	307	14.6	13.2–16.2
**Have you treated patients with a confirmed COVID-19 diagnosis?**	**2076**		
No or do not know	1766	85.1	83.5–86.5
Yes	310	14.9	13.5–16.5
**Have you suspected or tested yourself for COVID-19?**	**2120**		
No	1509	71.2	69.2–73.1
Suspect without test	251	11.8	10.5–13.3
Negative test	228	10.8	9.5–12.1
Inconclusive test	27	1.3	0.9–1.9
Positive test	105	4.9	4.1–6.0
**At present, which type of mask are you most frequently wearing for assisting patients?**	**2015**		
PFF2/N95	839	41.6	39.5–43.8
Disposable surgical mask	548	27.2	25.3–29.2
Surgical mask on top of PFF2/N95	414	20.6	18.8–22.4
Two disposable surgical masks	196	9.7	8.5–11.1
Washable fabric mask	18	0.9	0.1–1.4
**Main professional challenge during the pandemic**	**2041**		
Reduction of number of patients or financial gain	704	34.5	32.5–36.6
Fear of contracting COVID-19	701	34.4	32.3–36.4
Purchase or burden with new PPE	440	21.6	19.8–23.4
Reconciling work with household chores	133	6.5	5.5–7.7
Taking care of people with COVID-19	44	2.2	1.6–2.9
Update on scientific developments	19	0.9	0.1–1.5

^a^Varies from total N because of missing data for different questions

*CI* Confidence interval, *PPE* Personal protective equipment

### COVID-19 contraction rates among dentists and their main professional challenges

**Fig. 1:**
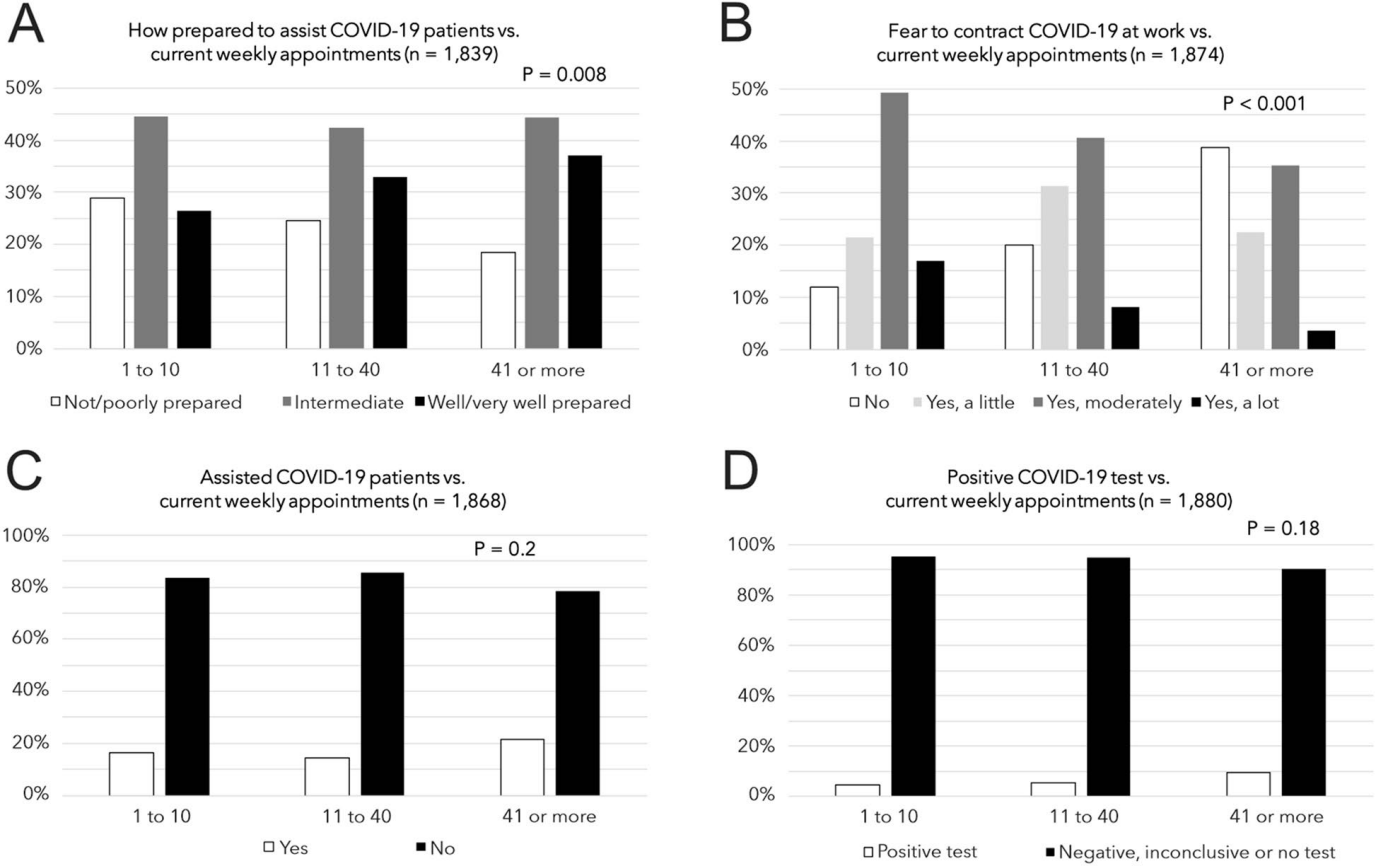
Graphs showing associations between the number of weekly patient appointments and **A**) how prepared the dentists reported themselves to be to assist patients with COVID-19; **B**) fear of contracting the disease at work; **C**) providing dental care assistance to COVID-19 patients; and **D**) positive COVID-19 testing by the dentists

Although 84.6% of respondents feared contracting the COVID-19 at work, only 17.0% indicated that they had been tested for the disease, with 4.9% reporting a positive test. The three main professional challenges faced by respondents were reduction in the number of patient appointments or financial gain (34.5%), fear of contracting COVID-19 at work (34.4%), and burden with new PPE or difficulty in purchasing PPE (21.6%). As shown in Fig. 1A, the number of weekly appointments was associated with how prepared the dentists reported themselves to be to assist patients with confirmed COVID-19. The fear to contracting COVID-19 at work was also influenced by the number of appointments (Fig. 1B): professionals assisting more patients a week, more often reported that they had no fear of contracting the disease, than dentists who were seeing fewer patients. The number of weekly appointments, however, was generally not associated with the report of having assisted COVID-19 patients (Fig. 1C) or with positive COVID-19 testing by the dentists themselves (Fig. 1D), but an upward trend in positive testing by dentists with increasing number of weekly appointments was noticed.

**Fig. 2:**
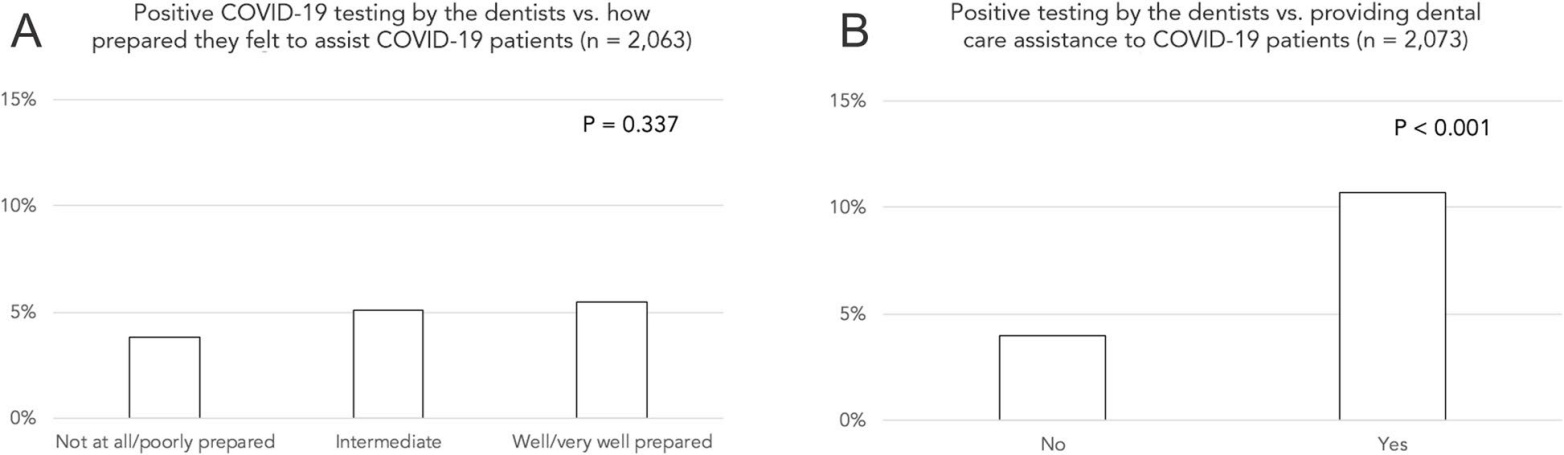
Graphs showing associations of positive COVID-19 testing by the dentists and **A**) how prepared they reported themselves to be to assist patients with COVID-19; **B**) report of having assisted COVID-19 patients. A positive association was observed in B

**Fig. 3:**
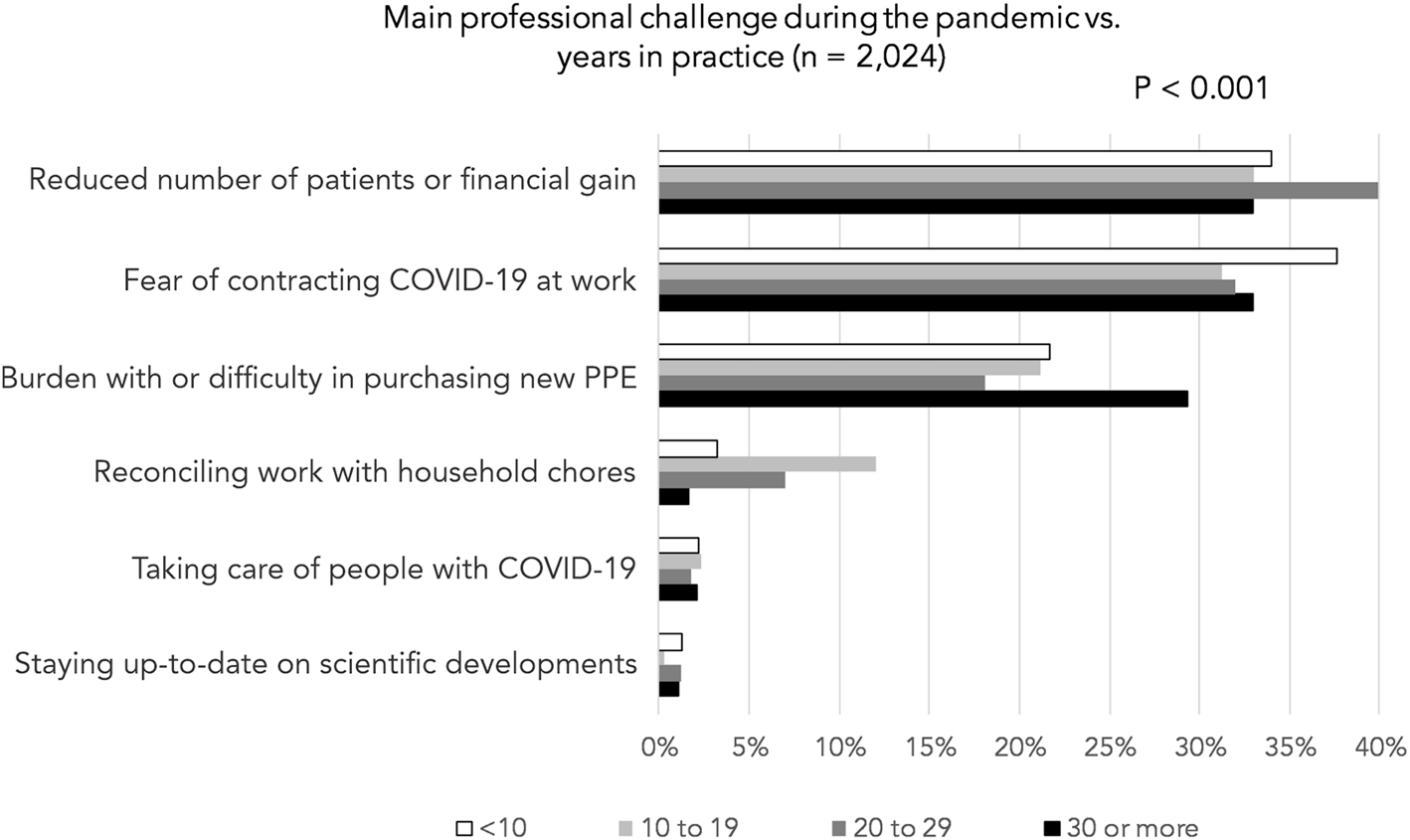
Main professional challenges faced during the pandemic reported by the respondents according to categories of years in practice (experience). Burden with the use of new PPE or difficulty in purchasing PPE was more often reported by professionals with 30 or more years in practice. Reconciling work with household chores was more common for professionals with an intermediate number of years of experience

No association was observed for positive COVID-19 testing by the dentists and how prepared they reported themselves to be to assist patients with COVID-19 (Fig. 2A). In contrast, a positive test by the dentists was associated with their reports of having assisted COVID-19 patients (Fig. 2B). Moreover, the clinical experience of the respondents (years in practice) influenced their report on the main professional challenges they faced during the pandemic (Fig. 3). Burden with the use of new PPE or difficulty in purchasing PPE was more often reported by professionals with 30 or more years in practice, whereas reconciling work with household chores was more common for professionals with an intermediate number of years in clinical practice.

### Dentists’ feelings about the pandemic

**Fig. 4:**
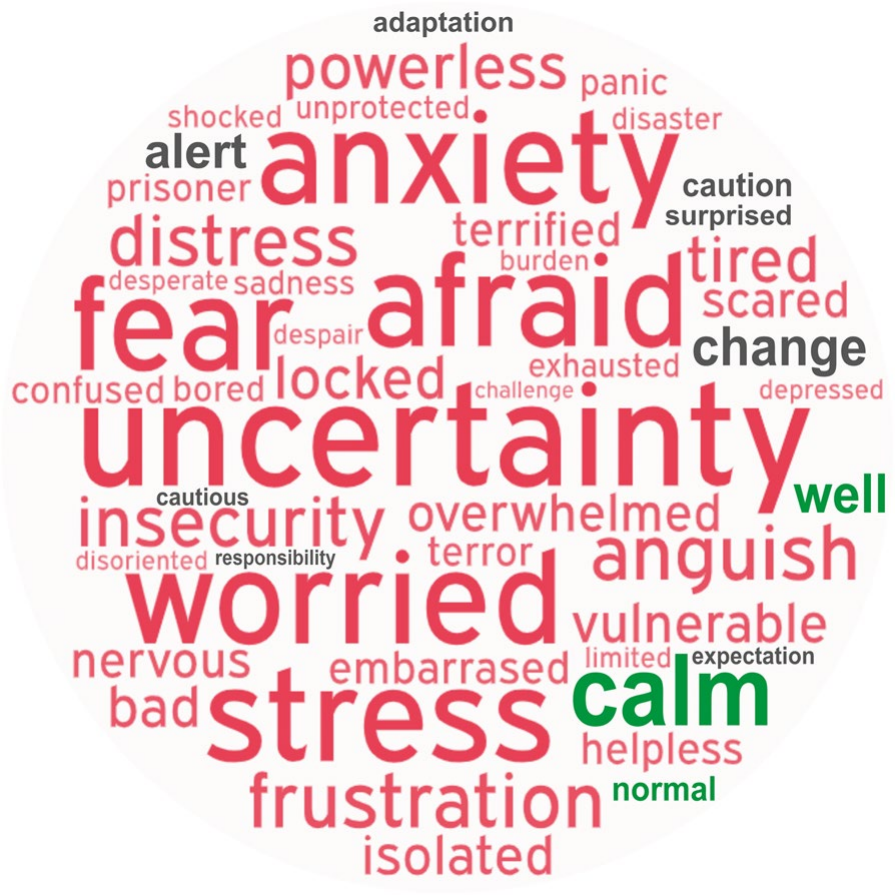
Word cloud showing the 50 most frequently mentioned words that the respondents associated with how they felt during the COVID-19 pandemic (wordart.com). The larger the font size, the more often the word was mentioned. Red, gray, and green fonts indicate negative, neutral, and positive feelings, respectively. Top 5 words cited were uncertainty (17.0%), afraid (13.1%), worried (6.8%), anxiety (6.0%), and stress (4.6%)

In the question asking respondents to cite a single word that they associated with how they felt during the COVID-19 pandemic (*n* = 2121), 235 different words were quoted. The top 5 most cited were uncertainty (17.0%), afraid (13.1%), worried (6.8%), anxiety (6.0%), and stress (4.6%). The 50 most frequent words are shown in a word cloud in Fig. 4. Table 3 presents results for the association between selected characteristics of the participating dentists and the feelings that they related with the pandemic (negative or neutral/positive feelings). Despite the overall massive prevalence of negative feelings, it was possible to observe differences. Females and dentists who felt poorly or not at all prepared to assist COVID-19 patients reported negative feelings more often. Negative feelings also were more prevalent for professionals who did not receive training for COVID-19 preventive measures, and for those reporting higher levels of fear to contract the disease at work. In contrast, the feelings were not associated with positive COVID-19 tests by the dentists or their report of having assisted COVID-19 patients.

**Table 3:** Associations between dentists’ characteristics and feelings related with the pandemic, Latin America, 2020 (*N* = 2127)

Variable/category^a^	Negative feeling		Neutral/positive feeling		*p*-value
	n	%	n	%	
**Sex (*n*** **= 2118)**					< 0.001
Female	1474	91.5	137	8.5	
Male	426	84.0	81	16.0	
**Preparation to assist COVID-19 patients (*n*** **= 2063)**					< 0.001
Not at all prepared/Poorly prepared	556	95.2	28	4.8	
Intermediately prepared	791	87.9	109	12.1	
Well/Very well prepared	502	86.7	77	13.3	
**Training for COVID-19 specific preventive measures (*n*** **= 2104)**					0.002
None	297	95.2	15	4.8	
Online training or general instructions	883	89.3	106	10.7	
Practical training	707	88.0	96	12.0	
**Fear to contract COVID-19 at work (*n*** **= 2096)**					< 0.001
No	259	80.4	63	19.6	
Yes, a little	459	89.1	56	10.9	
Yes, moderately	868	91.1	85	8.9	
Yes, a lot	293	95.8	13	4.2	
**Treated patients with COVID-19 (*n*** **= 2076)**					0.223
No or do not know	1586	90.0	177	10.0	
Yes	270	87.7	38	12.3	
**Positive COVID-19 test by the dentist (*n*** **= 2114)**					0.213
Negative, inconclusive, or no test	1798	89.5	212	10.5	
Yes	97	93.3	7	6.7	

^a^Varies from total N because of missing data for different questions

*P*-values from chi-square tests

## Discussion

This multi-country survey provided evidence on the impact of the COVID-19 pandemic on dental practice in Latin America, a region that has seldom been the focus of studies on the topic. Responses gathered by the end of 2020 from dentists practicing in 11 Spanish-speaking countries showed a reduction in the number of patient appointments and a high impact of the pandemic on clinical routines. Vaccines were still not available in the region at the time the study was conducted. As of June 2021, the proportion of fully vaccinated persons in the countries surveyed varied widely from 0 to 47% [22]. Considering the 11 countries in this study altogether, the average of the fully vaccinated population was 12% (median = 6.4%). Thus, although dentists and other healthcare professionals may have been prioritized for vaccination, there may still be a significant impact in place on the dental sector in the region.

Respondents reported that the lower volume of patients and fear of contracting the disease at work, were amongst the main professional challenges they faced during the pandemic. These two variables were found to be associated, i.e., more patients were assisted by professionals who had less fear of contracting COVID-19. By using the reported number of patients assisted before and during the pandemic, approximately 23,000 patients *per* week could have been missing dental appointments when considering this sample of respondents. If one assumes that the mean reduction was similar for other dentists in the countries surveyed, the overall figures of missing appointments would be overwhelming. However, care should be taken when extrapolating these findings, especially in a turbulent, quickly changing pandemic scenario. This study was conducted in the last quarter of 2020 and 85% of participants reported fear of contracting COVID-19 at work, which was similar to the 90% fear rate reported on May 2020 in Brazil [7], when the pandemic was just starting in Latin America and several countries around the globe faced lockdowns. It is noteworthy that these two studies used the same questionnaire, which was adapted to the present investigation. The present findings suggest that interval of 4–7 months between the survey in Brazil, the largest country in the region, and the present study were not enough to change the high rates of fear associated with working in Latin American dental offices. By comparison, COVID-19 patients had been seen by 5.3% of respondents in Brazil in May 2020, whereas this rate rose to 15% in the present survey, a difference that is likely to be explained by the escalation of the contagion curve in the meantime. As of June 2021, Latin America was still a significant pandemic epicenter. In the first 6 months of 2021, the number of deaths by COVID19 in all Latin America was higher than in 2020 [13]. Brazil has recorded the sad mark of 500 K deaths related to COVID-19, registering a 14-day moving average of 2058 deaths per day between June 7–20, 2021 [13]. Furthermore, in June 2021, the total death toll in the 11 countries surveyed surpassed the sad figure of 600 K deaths [13].

The prevalence of dentists who tested positive for COVID-19 was 4.9%, which was higher than the 2.6% rate reported in a cumulative analysis between June and November 2020 in the USA [23]. In October 2020, another survey in the USA reported that 3.1% of dental hygienists have tested positive or been diagnosed with COVID-19 [24]. In contrast, a study conducted in Italy between December 2020 and January 2021 reported a higher prevalence: 10.9% of the surveyed dentists reported to have had COVID-19, and 55% have had at least one family member or friend with the disease [25]. A later investigation in the Czech Republic, in June 2021, reported that 25% of surveyed dentists have had a positive COVID-19 test [26]. These varied prevalence rates among dentists addressed herein may be related to the different assessment periods, distinct public health measures in response to the pandemic in different countries, and distinct SARS-CoV-2 spreading rates. Although the end of the pandemic was not in the radar of dentists when the present study was conducted, the situation may be different in 2022. A recent article suggested that the unprecedent global levels of infection with the omicron variant of SARS-CoV-2 may potentially lead to the end of the pandemic and the era of exceptional measures to control the disease [27].

The survey conducted in Italy [25] also showed that 82% of the dental care providers had a positive intention to be vaccinated against COVID-19, whereas 18% had a negative intention. A study with a sample of students enrolled in an international dental association reported that 34.9% of respondents disagreed or were hesitant about taking COVID-19 vaccines [28]. In addition to the high level of vaccine hesitancy observed among students, the study also showed that social media, mistrust of governments, and insufficient knowledge about vaccines and their safety were potential barriers for vaccination [28]. Vaccine hesitancy also has been observed among dental and medical undergraduates in the USA in a study suggesting the need for school curricula to enhance student knowledge about vaccines [29]. A study in the UK addressing COVID-19 seroprevalence and response to vaccines in dental care professionals suggested that natural infection alone is unlikely to generate durable herd immunity, and showed that vaccination was associated with an antibody response indicative of immunological memory [30].

Positive COVID-19 test reported by the dentist was not associated with the weekly number of patients seen by the professional, which could suggest that preventive measures in offices and PPE use have been effective in preventing contamination. Roughly two thirds of participants indicated that PFF2/N95 were the masks most often used, a relatively low prevalence because PFF2/N95 masks offer better protection than surgical masks, and the dental office is a high-risk environment. The low level of use could be linked to difficulties in purchasing PPE and burden-associated discomfort with their use, as mentioned by 22% of the respondents. However, a positive test was associated with the report of having assisted one or more patients with a diagnosis of COVID-19. This finding could be related to the risk of infection by SARS-CoV-2 in the clinics, however, at the same time, related to a higher prevalence of COVID-19 testing among professionals that have assisted patients with the disease. Massive testing has been at the center of discussions to control the spread of SARS-CoV-2. Varied molecular and serologic assays have been developed and implemented worldwide, but there has been variability in quality and accuracy across different tests [31]. The respondents of the present survey were not asked about which test they relied on for a positive diagnosis, and it is known that the highly accurate reverse transcription polymerase chain reaction tests are costly and have a slow turnaround time. Cheaper, quicker tests may have variable false positive/negative results that could play a role in the event that they were used for the diagnosis. Dental care professionals are advised to keep up with strict, effective preventive measures at the offices [32] to reduce their chances of contamination while the outbreak is not yet under control in Latin America.

When respondents used a single word to refer to their feelings about the pandemic, a massive prevalence of bad feelings was observed. To the best of our knowledge, this is the first study to use this type of approach to create a picture of how dentists feel in the ongoing pandemic. The scenario of case and death counts attributable to COVID-19 in Latin America [13] and the absence of vaccines at the time the survey was carried out may explain why uncertainty topped the list of feelings cited, followed by other associated feelings including fear, worry, anxiety, and stress. Not surprisingly, higher levels of fear to contract COVID-19 were associated with more frequent negative feelings. In addition, negative feelings were more often reported by females, dentists who did not receive training for preventive COVID-19 measures, and those who did not feel well prepared to assist COVID-19 patients. Phycological issues in healthcare workers have been the scope of several studies since the pandemic was declared. A study during a lockdown period indicated substantial psychological burden among dental personnel in terms of fear of being infected or infecting others, with a higher impact on females and less experienced clinicians [11]. Health professionals were reported to be at high risk of incurring burnout conditions during the COVID-19 emergency [33]. An investigation suggested that raising the psychological resilience of healthcare professionals should address their quality of sleep, positive emotions, and life satisfaction [34]. The importance of investing resources in research, prevention, and treatment to promote mental health of healthcare professionals has been debated [35] in addition to facing the long-term mental health consequences of the outbreak [33]. Since vaccines were not available in the region in the period when the survey was implemented, further investigation could evaluate how vaccination has influenced the presence of fear in dental offices throughout Latin America.

Multivariable regression analysis was not used in this study since its purpose was descriptive, and controlling for confounding would be neither appropriate or necessary [36]. However, the study has limitations that should be acknowledged, and care should be taken when extrapolating the presented findings. One limitation in the recruitment strategy involved the self-selection bias, in which individuals were free to accept or not to participate in the survey and researchers could not control the self-selection process. The combination of email and social media recruitments aimed to improve response rates and lead to a more diverse population in a multi-country scenario [21]. Although most respondents were reached by emails and messages sent to registered professionals, and the open campaign on social media explicitly targeted dentists, no strategy was used to verify whether the participants were actual dental practitioners. These aspects could have led to a higher chance of recruiting participants who were more concerned with the pandemic or more willing to cooperate with sanitary measures, or even participants who were not dentists. In addition, the self-reported responses about the number of patients assisted before the pandemic could be influenced by recall bias, meaning that the memories could be affected by posterior events and experiences. In further studies, data on weekly appointments could be more accurate if the information were collected by using dental records, for instance. A strength of the study was that a large sample of dentists were recruited from a region that is still struggling to deal with the pandemic. In addition, the study suggests that WhatsApp and emails are important tools in contemporary online surveys since these invitations accounted for over 80% of the participation.

In conclusion, this study with dentists working in 11 Latin American countries suggested a high impact of the COVID-19 pandemic on professional practices and clinical dental care routines. The self-reported prevalence of positive COVID-19 tests seemed to be low in the sample (4.9%), which reported a high prevalence of fear of contracting the disease at work (85%). A massive prevalence of bad feelings associated with the pandemic was expressed by the respondents. Further investigation on the impact of vaccination to the dental sector in Latin America is warranted.

## Supplementary Information

**Additional file 1.**

## Data Availability

The datasets generated and/or analyzed during the current study are available in the Open Science Framework repository, https://osf.io/dnbgs/.

## Abbreviations

COVID-19: Coronavirus disease
PPE: Personal protective equipment
SARS-CoV-2: Severe acute respiratory syndrome coronavirus 2
CROSS: Consensus-based Checklist for Reporting of Survey Studies
SD: Standard deviation
