# Supplementary material for: A multi-country survey on the impact of COVID-19 on dental practice and dentists’ feelings in Latin America
Source: BMC Health Serv Res. 2022 Mar 25;22:393. doi: 10.1186/s12913-022-07792-y (PMC8951658; doi:10.1186/s12913-022-07792-y)
Supplement: Supplementary file 1 — Additional file 1. [file 12913_2022_7792_MOESM1_ESM.docx]

**Questionnaire (original language: Latin American Spanish)**

URL: https://forms.gle/hBx2vkQSu6CCWjpS9

The following invitation message was used:

“Dear colleague,

You are being invited to participate in this multi-country project "Impact of COVID-19 on dentistry: multicenter study with dentists from Latin America", which aims to evaluate the impact of the pandemic on dental practice. Your participation will be responding an online questionnaire containing questions about your clinical routine.

The project involves the application of the questionnaire to dentists from different Latin American countries. All information related to the project, including the questionnaire itself, is available after clicking on the "start survey" button shown below.

Your participation is very important to us, thank you very much.

START SURVEY”

| Impact of COVID-19 on routine of dental care assistance  Dear fellow dentist,  The clinical treatment of patients during the pandemic can put dentists in situations that involve a high risk of COVID-19 contagion. This situation has required clinical routine adaptations to be undertaken; understanding the impact of these new routines can help to improve dental care assistance.  You are invited to participate in this multicenter project “Impact of COVID-19 on dentistry: a multicenter study with dentists in Latin America”. The objective is to assess the impact of the pandemic on dental practice. Your participation will involve completing this online survey about your clinical routine. If you do not want to participate, please leave the page or simply do not finish the survey. If you are not a dentist or did not graduate from dental school yet, we ask you not to participate. If you already answered this survey, we thank you for your participation.  Your participation is voluntary; no identification is necessary. If you agree, at the end of the survey, you can provide your email address to participate in a new round of the same survey. In this case, we guarantee that your data and responses will be treated confidentially and anonymously. There is no remuneration for your participation in this survey.  Potential benefits of this research include identification of the pandemic impact on the dental practice and prospection of solutions to help improve clinical routines. In this sense, the results will be published in scientific articles and social media posts.  Potential risks of this research involve discomfort answering any given question. In this case, the option “I'd rather not declare’ will be available. The option “Does not apply” can be used for questions you think do not apply to your case. Like any activity carried out over the Internet, there is a possibility that the information collected may be hacked or accessed by the owner of the server. However, as the probability of this happening is low and no personal data is requested, this poses low risk to the investigation.  If you agree to participate in the survey, this page will be your Informed Consent Form. To maintain a copy, print this page or save it as a PDF file. If you need any clarification about this research or want to access additional information, please contact the investigators (information provided below). If you prefer, you can contact us before answering the survey. You can contact us after submitting your questionnaire if you want to withdraw your participation consent.  If you agree to participate, we ask that you answer the survey completely. The average response time is 7 (seven) minutes.  **YOUR PARTICIPATION IS VERY IMPORTANT!**  Institution and researchers  Prof. Rafael Moraes (principal investigator) – rafael.moraes@ufpel.edu.br  Graduate Program in Dentistry  Universidade Federal de Pelotas  Rua Gonçalves Chaves 457  96015-560 Pelotas, RS, Brasil  Prof. Andrés Dávila Sánchez - cadavilas@usfq.edu.ec  Universidad San Francisco de Quito, Ecuador  Prof. Carlos Enrique Cuevas Suárez - cecuevas@uaeh.edu.mx  Universidad Autónoma del Estado de Hidalgo, México  Prof. Eduardo Fernández Godoy - eduardo.fernandez@uautonoma.cl  Universidad Autónoma de Chile / Universidad de Chile, Chile  Prof. Guillermo Grazioli - ggrazioli@odon.edu.uy  Universidad de la República, Uruguay  Prof. Luis Alfonso Arana Gordillo - luis.arana00@usc.edu.co  Universidad Santiago de Cali, Colombia  Prof. Luis Felipe Rondon - rondonluisfelipe@gmail.com  Universidad de Los Andes, Venezuela  Profa. María Raquel Fernández Morínigo - mafernandez@uaa.edu.py  Universidad Autónoma de Asunción, Paraguay  Profa. Patricia Grau Guillón - p.grau@prof.unibe.edu.do  Universidad Iberoamericana, República Dominicana  Profa. Tania Mercedes López Martínez - tlopez@unan.edu.ni  Universidad Nacional Autónoma de Nicaragua, Nicaragua  Prof. Wilfredo Gustavo Escalante Otárola - wilfredoescalante@gmail.com  Universidad Nacional Jorge Basadre Grohmann, Perú  Dr. Willy Bustillos Torrez - willybustillos4@gmail.com  Bolivia  Research Ethics Board information:  School of Medicine  Universidade Federal de Pelotas, Brasil  Phone: +55 53 3301.1801  Do you agree to participate in the study voluntarily?  0 – No  1 – Yes | |
| --- | --- |
| **Section 1: Questions related to your professional profile** | |
| 1. What is your current country of residence? | 0 – Bolivia  1 – Brazil  2 – Ecuador  3 – Chile  4 – Colombia  5 – Mexico  6 – Nicaragua  7 – Paraguay  8 – Peru  9 – Dominican Republic  10 – Uruguay  11 – Venezuela  12 – I'd rather not declare  Other: |
| 1. By what means did you receive the invitation to participate in this survey or saw an invitation being posted? Respond relative to the first time that you saw or received the invitation. | 0 – Email  1 – WhatsApp message  2 – Instagram post or message  3 – Facebook post or message  4 – Twitter post or message  5 – I don’t know  6 – I'd rather not declare  Other: |
| 1. Who sent you the invitation to participate in the survey or who posted the invitation on a social network? Respond relative to the first time you saw or received the invitation. | 0 – Dental council of my country, Ministry of Health or professional entities  1 – A fellow dentist  2 – A professor or investigator  3 – A university or scientific profile on social media  4 – I don’t know  5 – I'd rather not declare  88 – Does not apply  Other: |
| 1. Why did you decide to participate in this survey? Please indicate the main reason | 0 – Contributing to my country during the pandemic or to dentistry in general  1 – Trying to get up-to-date information on the pandemic  2 – Because I already have a habit of participating in surveys  3 – Because I believe in the ability of science to generate knowledge about the pandemic  4 – Because I am afraid of contracting COVID-19  5 – To honor the colleague or dental organization who/that sent me the invitation  6 – I don’t know  7 – I'd rather not declare  88 – Does not apply  Other: |
| 1. What is your sex? | 0 – Male  1 – Female  2 – I’d rather not declare |
| 1. How old are you? | I’d rather not declare  List of ages: from 18 – 80 or + |
| 1. From which type of institution did you graduate in Dentistry? | 0 – I did not graduate in Dentistry  1 – Public  2 – Foreign diploma revalidated  3 – Private  4 – I don’t know  5 – I’d rather not declare  Other: |
| 1. In which year did you graduate in Dentistry?   *Attention: if you are not a dentist or did not graduate yet, please do not answer this survey* | I’d rather not declare  List of years: from 2020 to 1960 or before |
| 1. In which type of service do you work mostly?   *If you work in more than one sector, choose the one you consider your primary workplace.* | 0 – Dentist in public network  1 – Dentist in private network  2 – Teaching  3 – Management  4 – I don’t know  5 – I’d rather not declare  88 – Does not apply |
| 1. Have you completed postgraduate education in Dentistry?   *Please select the highest degree* | 0 - No  1 – Yes, short term courses  2 – Yes, residency or special advanced training  3 – Yes, Masters  4 – Yes, PhD  5 – I’d rather not declare |
| 1. Before the pandemic, how many patients did you assist, in average, in a complete work week? | I don’t know  I’d rather not declare  Does not apply  List of numbers: 1 to 130 or + |
| **Section 2: Questions related to your professional practice during the pandemic** | |
| 1. What has been your main professional challenge during the pandemic? | 0 – Fear of contracting COVID-19 at work or when going to work  1 – Difficulty purchasing personal protective equipment  2 – Inconvenience in the use of new personal protective equipment  3 – Reduction of the number of patients  4 – Reduction of financial gain or salary  5 – Difficulty in updating on scientific developments in relation to the pandemic  6 – Difficulty in reconciling work with household chores and/or childcare  7 – Taking care of people who contracted COVID-19  8 – I don’t know  9 – I’d rather not declare  88 – Does not apply  Other: |
| 1. How do you describe your current work status? | 0 – I’m working as usual  1 – I’m working, but less frequently  2 – I’m assisting emergencies only  3 – I’m not working  4 – I’d rather not declare  88 – Does not apply |
| 1. How do you rate the impact of the pandemic in your work routine? | 0 – There was no impact  1 – Low impact  2 – Intermediate impact  3 – High impact  4 – Very high impact  5 – I’d rather not declare  88 – I do not have a clinical routine or does not apply |
| 1. In average, how many patients are you currently assisting in a complete work week? | I don’t know  I’d rather not declare  I do not work with clinics or does not apply  None, because of the pandemic  None, due to other reasons  List of numbers: 1 – 130 or + |
| 1. Have you received any training on COVID-19 preventive measures? | 0 – No  1 – Only general instructions  2 – I have received practical training  3 – I’d rather not declare  88 – Does not apply |
| 1. Which preventive measures did you receive training on?   *Mark all that apply* | 0 – I did not receive any training  1 – Use of personal protective equipment  2 – Preparation of the office before appointments  3 – Preparation of the office between appointments  4 – COVID-19 screening in patients  5 – Infection control in the workplace  6 – I’d rather not declare  88 – Does not apply  Other: |
| 1. How prepared do you feel to assist patients with confirmed diagnosis of COVID-19? | 0 – Not at all prepared  1 – Poorly prepared  2 – Intermediately  3 – Well prepared  4 – Very well prepared  5 – I’d rather not declare  88 – Does not apply |
| 1. Which of the following personal protective equipment (PPE) are available in your work place?   *Please select all that apply* | 0 – Surgical mask  1 – Disposable cap  2 – Fabric cap  3 – Disposable surgical coat  4 – Reusable surgical coat  5 – PFF2 (N95) mask  6 – Face shield  7 – Protective goggles  8 – Surgical scrub  9 – I’d rather not declare  88 – Does not apply  Other: |
| 1. At present, which type of mask are you most frequently wearing for assisting patients? | 0 – Disposable surgical mask  1 – Reusable fabric mask  2 – PFF2 (N95) mask  3 – Surgical mask over PFF2 (N95) mask  4 – Two disposable surgical masks  5 – I’d rather not declare  88 – Does not apply  Other: |
| 1. Have you assisted any patients via online appointment during the pandemic? | 0 – No, and I’m not willing to make them  1 – No, but I’m willing to make them  2 – Yes, and I rate the experience as positive in general  3 – Yes, but I rate the experience as negative in general  4 – I’d rather not declare  88 – Does not apply |
| **Section 3: Structure at your workplace** | |
| 1. Did changes in your clinical routine due to COVID-19 result in additional costs? | 0 – No  1 – Yes, but treatment prices were not adjusted for patients  2 – Yes, and treatment prices were adjusted for patients  3 – I’d rather not declare  88 – Does not apply |
| 1. Which of the following sources for updated information on professional conduct toward the pandemic do you access? | 0 – I do not access information  1 – Official bodies (e.g. Ministry of Health, Dental Council)  2 – Universities or research centers websites  3 – Scientific literature  4 – Health websites/blogs  5 – Social network services  6 – Fellow dentists  7 – I’d rather not declare  Other: |
| 1. Do you perform any type of patient screening concerning to COVID-19 before appointments in your main workplace?   *Mark all that apply* | 0 – No, I am using normal anamnesis  1 – Yes, specific questionnaire for COVID-19  2 – Yes, temperature check of patients  3 – Recommend mouthwashes with antimicrobials  4 – I Prefer not to declare  88 – Does not apply  Other: |
| 1. Do you currently fear to contract COVID-19 at work? | 0 – No  1 – Yes, a little  2 – Yes, moderately  3 – Yes, a lot  4 – I don’t know  5 – I’d rather not declare  88 – Does not apply |
| 1. Have you assisted patients with confirmed diagnostics of COVID-19? | 0 – No  1 – I don’t know  2 – Yes  3 – I’d rather not declare  88 – Does not apply |
| 1. Have you suspected or tested yourself for COVID-19? | 0 – No  1 – Suspicion without test  2 – I tested negative for COVID-19  3 – My test was inconclusive for COVID-19  4 – I tested positive for COVID-19  5 – I’d rather not declare |
| 1. Do you agree with social distancing measures adopted currently in your city? | 0 – Fully disagree  1 – Partially disagree  2 – Not agree or disagree  3 – Partially agree  4 – Fully agree  5 – I’d rather not declare  88 – Does not apply |
| **Final section** | |
| 1. With just ONE WORD, describe the feeling you associate today with how you felt during the COVID-19 pandemic: | Open question |
| 1. Have you participated before in a survey on the impact of COVID-19 on dentistry? | 0 – No  1 – Yes  2 – I don’t know  3 – I’d rather not declare |
| 1. What type of incentive could motivate you to participate in a second phase of this investigation in the future? | 0 – Financial incentive  1 – Priority access to data from the first phase  2 – Receiving scientific articles originated from the investigation when published  3 – Receiving partial reports on the progress of the investigation  4 – Receiving the invitation from people I like or trust  5 – Knowing how many dentists in my country participated in the first stage  6 – In case the incidence or mortality of the pandemic increases in my country  7 – I don't need any incentive to participate in the next stage  8 – I don’t know  9 – I’d rather not declare |
| This study will have second phase. If you agree, please leave your email address so you can receive our communication in the future. Your email address will not be disclosed in any way and your answers will not be associated to your email (investigators’ confidentiality commitment). | Your e-mail: |
| THANK YOU VERY MUCH FOR YOUR PARTICIPATION! | |
